# Nanomedicine-Mediated Autophagy Modulation in Placental Impairment Versus Cancers: A Narrative Review

**DOI:** 10.3390/pharmaceutics18070809

**Published:** 2026-06-30

**Authors:** Melinda Ildiko Mitranovici, Viviana Ivan, Adrian Apostol, Liviu Moraru, Septimiu Voidazan, Raluca Niculescu, Ioana Cristina Rotar, Florin Bobirca, Andreea Taisia Tiron, Laura Georgiana Caravia

**Affiliations:** 1 Department of Obstetrics and Gynecology, Emergency County Hospital Hunedoara, 331057 Hunedoara, Romania; mitranovicimelinda@yahoo.ro; 2 Doctoral School of Medicine and Pharmacy, George Emil Palade University of Medicine, Pharmacy, Science, and Technology of Targu Mures, 540142 Targu Mures, Romania; 3 Department of Cardiology, “Victor Babes” University of Medicine and Pharmacy, 2 Eftimie Murgu Sq., 300041 Timisoara, Romania; adrian.apostol@umft.ro; 4 Department of Anatomy, University of Medicine, Pharmacy, Sciences and Technology “George Emil Palade”, 540142 Targu Mures, Romania; liviu.moraru@umfst.ro; 5 Department of Epidemiology, “George Emil Palade” University of Medicine, Pharmacy, Sciences and Technology, 540142 Targu Mures, Romania; septimiu.voidazan@umfst.ro; 6 Department of Pathophysiology, “George Emil Palade” University of Medicine, Pharmacy, Science, and Technology of Targu Mures, 38 Gheorghe Marinescu Street, 540142 Targu Mures, Romania; raluca.niculescu@umfst.ro; 7 Department of Obstetrics and Gynecology I, Mother and Child Department, “Iuliu Hatieganu” University of Medicine and Pharmacy, 400012 Cluj-Napoca, Romania; cristina.rotar@umfcluj.ro; 8 Faculty of Medicine, “Carol Davila” University of Medicine and Pharmacy, 050474 Bucharest, Romania; bobirca@umfcd.ro (F.B.); taisia_andreea@yahoo.com (A.T.T.); laura.caravia@umfcd.ro (L.G.C.)

**Keywords:** autophagy, pregnancy disorders, cancer stem cells, nanotechnology

## Abstract

The biggest challenge faced by classical anticancer therapy is drug resistance, which causes cancer recurrence and metastasis. Two underlying mechanisms could be responsible, including the stemness of pro-survival autophagy-associated cancer stem cells (CSCs). **Background/Objectives**: The relationship between CSCs and autophagy in gynecological cancer is still unknown. However, it has been shown that CSCs’ in vitro self-renewal ability is decreased when autophagy is inhibited. Helping to maintain normal tissue homeostasis, autophagy is a catabolic process involved in degrading long-lived proteins and cytoplasmic organelles. Autophagy acts as a key player in the human body’s self-regenerating tissues. It also has a reproductive function, contributing to decidualization for a successful pregnancy. The aim of our review is to identify similarities and differences between these processes, using these findings to discover new therapeutic strategies through nanotechnology. **Method**: We conducted a narrative review, identifying heterogeneity in the data in the literature, and found 153 relevant articles. **Discussions**: While autophagy has been proven to be capable of acting as a tumor suppressor, it also promotes tumor progression. Moreover, it has been linked to cancer stem cell regulation, therapy resistance, cancer invasion, and metastasis. Several molecular mechanisms have been linked to autophagy. Remarkably, some cellular processes required for proper placentation, including autophagy, are common between placental development and tumor growth. Just as trophoblast cells invade and migrate, so do cancer cells. While in the trophoblast, this phenomenon is programmed and controlled; in cancer, this regulation is lost. As shown, we thus observed commonalities and discrepancies in the phenotypes and underlying molecular mechanisms of autophagy regulation in preeclampsia versus cancer contexts. Translational applicability of nanomedicine research strategies and design paradigms between preeclampsia intervention and cancer therapy has been sought. **Conclusions**: Autophagy-based nanotechnology seems to be feasible in both placental ischemia in preeclampsia and cancers. This review draws parallels between targeted treatments in malignancies and placenta-derived PE. Comparing these diseases provides a novel molecular rationale and the possibility of identifying treatment through autophagy modulation.

## 1. Introduction

Autophagy is a catalytic process involving damaged proteins and organelles that takes place in lysosomes under normal conditions. It maintains homeostasis and is essential for cell survival during hypoxia and starvation and in vascular remodeling [1,2]. Autophagy can be divided into three types: micro-autophagy, characterized by the direct uptake of damaged proteins and organelles through lysosomal invagination; chaperone-mediated autophagy [3,4]; and macro-autophagy, which involves autophagosomes through autophagic cargo delivery to lysosomes by membrane fusion, which is called an autophago-lysosome [5].

In cancer cells, depending on the stage and tumor considered, autophagy has a dual role both as a tumor promoter and inhibitor [1,2]. In the case of tumor progression, autophagy is linked to the epithelial-to-mesenchymal transition (EMT), as well as tumor microenvironment (TME) modulation [1]. Some of the molecular mechanisms required to support tumor progression share common characteristics with placental development. Both the placenta and cancers involve invasion, proliferation, and migration. The difference lies in the fact that, in the former, this process is limited in time and space, while in cancer, any such regulation is lost [6,7]. Autophagy in the placenta is essential in vascular remodeling, as well as in the degradation of damaged proteins and organelles [1,2].

Cancer researchers have lately focused on conventional treatment resistance, which has two underlying mechanisms: cancer stem cell (CSC) stemness and pro-survival autophagy activation [6], the relationship between which remains unknown [1]. Tumors’ tendency towards recurrence and metastasis is due to traditional cancer therapy failure, based on cancer stemness cells (CSCs). CSCs have an inaccessible nature; they usually reside in hypoxic TME and are characterized by high resistance to conventional treatments such as chemotherapy, radiotherapy, and surgery. Considerable progress has been achieved in nanotechnology with the design of organic and inorganic nanoplatforms [7,8,9]. However, antitumor therapeutic strategies that target autophagy are scarce [10]. For now, a few potential NPs have been developed for existing autophagy modulatory compounds [10,11].

The aim of our review is to identify similarities and differences between these processes in order to use these findings in uncovering new therapeutic strategies through nanotechnology.

## 2. Materials and Methods

The main question concerns the usefulness of autophagy targeting in placenta-related pathologies versus cancer treatment. There is heterogeneity in the data in the literature, as these two separate pathologies both involve proliferation and invasion, with autophagy playing an important role. For this reason, a narrative review is the most appropriate. We included a wide range of studies, highlighting novelties. Based on specific keywords, a thorough search was conducted in Google Scholar, Cochrane Database and PubMed, from which we extracted 10,700 titles. We excluded case reports, books, editorials and reports from our review; inclusion criteria were full-text articles written in English and informative studies with an appropriate study design. Duplicates and articles for which only the abstract was available were also excluded, leaving 153 titles.

Our manuscript was structured into sections about autophagy characteristics in the placenta and its pathologies, autophagy in cancers, treatment options targeting autophagy in placenta-related diseases and in cancers, and well as the use of nanotechnology in these contexts.

## 3. Placental Ischemia- and Preeclampsia-Associated Autophagy

According to the American College of Obstetricians and Gynecologists, preeclampsia (PE) is a multisystemic disorder characterized by persistent hypertension (≥140/90 mmHg) with or without proteinuria (≥300 mg in urine collected over 24 h) and other maternal complications after 20 weeks of gestation [12]. Although a complete understanding of PE remains elusive, its pathogenesis is linked to the placental ischemia [13]. The only current curative treatment is placenta removal and fetus delivery [14,15]. For now, current guidelines recommend only antihypertensive agents (labetalol, methyldopa and/or nifedipine), although the prophylactic administration of a low dose of aspirin (150 mg/day) is accepted before 16 weeks of gestation [15].

Autophagy has an important role in placental development [12,16,17]. In healthy pregnancies, oxidative stress is increased during the first trimester, but this is exacerbated in preeclampsia (PE). In PE, free oxidative radicals are released, which induce endothelial damage [15]. Pro-inflammatory factors are produced under hypoxic conditions, leading to senescence in trophoblast and placental ischemia [18,19]. Ischemic lesions affect mitochondrial respiration, meaning that a vicious circle is established that leads to impaired autophagy [20,21]. Over-activated autophagy induces cellular death known as programmed cell death type II, while in apoptosis, this is programmed cell death type I. Autophagy has a dual role in either activating or inhibiting cellular programmed death. Autophagy can increase in preeclampsia and intrauterine growth restriction, but the mechanisms of this are still unclear [16]. Mitochondria are responsible for nutrient transport and energy-producing oxidative phosphorylation, processes essential for both placental growth and optimal function [22,23]. They are also involved in apoptosis through excessive reactive oxygen species (ROS) generation, followed by oxidative stress and mitochondrial DNA (mtDNA) damage [24,25,26].

Although autophagy’s mechanism of action is not completely understood, genes responsible for this process have been identified. In dysfunctional cells, autophagy-related gene 3 (*Atg3*) and *Atg17* lead to autophagy activation under stress signals from the mammalian target of rapamycin (mTOR) complex 1 [16]. The phosphatidylinositol-4,5-bisphosphate 3-kinase (PI3K) complex type 3 is activated during this process, resulting in autophagosome formation [27,28]. Then, the fusion of an autophagosome and a lysosome leads to the former digesting the latter in the cell death-promoting degradation step [29]. Various signaling pathways are involved, including PI3K/AKT/mTOR and AMPK/mTOR [1]. However, the most important inducing factor for trophoblastic autophagy is excessive hypoxia [30], which can lead to placental cellular damage and endothelial cell dysfunction [15]. Hypoxia induces HIF-1α overexpression and light chain protein (LC3-II) activation, as well as repressing phosphorylated mTOR [31]. HIF-1α and HIF-2α are the mediators of the hypoxic stress signal [13,32,33]. Light chain protein (LC3), beclin-1, and the mammalian target of rapamycin (mTOR) are direct markers of autophagy; they decrease under hypoxic conditions [13,34] and their activation leads to placental impairment [12,34]. HIF activates autophagy through BNIP3 (Bcl-2/E1B 19 kDa-interacting protein 3) [35]. These markers should be evaluated in the placentas of pregnant women with PE and IUGR [12,16]. Autophagy activation leads to increased matrix metalloproteinase (MMP)-2 and MMP-9 expression, which in turn leads to trophoblast invasion, a normal process during pregnancy [16]. Autophagy inactivation causes poor vascular remodeling due to excessive hypoxia, which is observed in preeclampsia [36,37], causing vascular endothelial growth factor A (VEGFA) downregulation and FMS-like tyrosine kinase-1 (Flt1) upregulation [37]. mTOR inhibition activates the NF-κB in the inflammatory pathway via HIF, leading to soluble Fms-like tyrosine kinase-1 (sFlt-1) release followed by maternal vascular damage [13].

In Li et al.’s study, hypoxia-induced autophagy affected apoptosis and mRNA and VEGFA protein expression; furthermore, LC3B protein expression was lower in placentae from those with early onset PE compared to the control group [37]. Furthermore, in Weel et al.’s study of 40 pregnant women (20 with PE and 20 with normal pregnancies), mTOR gene expression was significantly higher, while the mRNA for LC3 and beclin-1 was significantly lower in the placenta of those with PE compared to the control group [12].

Ribeiro et al. observed p62/SQSTM1 expression, which is seemingly an autophagy marker. They observed that p62/SQSTM1 mRNA levels were significantly lower, while protein expression was significantly higher, in the placentas of pregnant women with PE (no = 20) compared to normal equivalents (no = 20) [17].

In Zhou et al.’s study, Bcl-2/adenovirus E1B 19 kDa interacting protein 3 (BNIP3) was investigated to prove its association with PE. BNIP3 is a mediator of mitophagy; its suppressed expression and oxidative stress have been seen in PE patients [38]. Wan identified and validated PKM, LEP, and HK2 as autophagy-related genes in PE, which is also associated with metabolic pathways, the immune microenvironment and reduced M2 macrophages [39].

## 4. Autophagy in Cancers Versus Placental Development

Autophagy in cancer progression has a dual role; its activation can either promote cancer cell survival or suppress tumors, depending on the context. This process happens under nutrient deprivation and hypoxia [10].

Interestingly, autophagy activation is a key process in both placental and cancer development [33,40], but its mechanism remains controversial [41]. The development of both cancer and trophoblast cells involves different molecules such as hormones, growth factors, oncogenes, receptors, and proteins, which mediate their proliferation, invasion capabilities and metastasis [42]. Low oxygen levels upregulate hypoxia-inducible factor (HIF), leading to immunomodulation and angiogenesis [43], the latter being another major process involved in both trophoblast and cancer development [44,45]. Vascular endothelial (VEGF) and fibroblast growth factors (FGF) are specific proteins that are regulated by hypoxia, are secreted by the placenta and in cancer, and that stimulate angiogenesis. Angiostatin and fibronectin are proteins that inhibit angiogenesis and that seem to be influenced by autophagy [1,46]. Hypoxia-induced autophagy in cancer is an adaptive response promoting tumor survival and therapeutic resistance. In preeclampsia, adaptive autophagy in early pregnancy under normal hypoxic conditions transitions to an impaired autophagy–lysosomal mechanism, leading to excessive trophoblast stress. We elaborate on the commonalities and discrepancies in the phenotypes and underlying molecular mechanisms of hypoxia-mediated autophagy regulation in preeclampsia versus cancer contexts [47,48,49].

### 4.1. Commonalities in Molecular Mechanisms

- In both environments, cancer and PE, hypoxia-inducible factor 1-alpha HIF-1α is a key regulator, activating autophagy-related genes to cope with low oxygen levels [47,49].
- Hypoxia triggers autophagy by suppressing the mTOR pathway and activating AMPK recycling damaged proteins and cell components [48].
- Both diseases use BNIP3-Mediated Mitophagy to induce mitochondrial autophagy, which reduces reactive oxygen species (ROS). This process prevents apoptosis under low-oxygen conditions [48].

### 4.2. Discrepancies in Phenotypes and Molecular Mechanisms

In cancer, adaptive high autophagy is observed, which supports rapid growth. Conversely, in early-onset PE, vascular impairment is found with a chronic hypoxia-reoxygenation mechanism, followed by autophagy–lysosomal machinery impairment with a decrease in mature autophagosomes and the accumulation of damaged components [48,49].

Preeclampsia is characterized by the accumulation of p53, which is a substrate of autophagy, indicating a blockage in the autophagy pathway, while in cancers, p53 is degraded, resulting in recycling of cellular components [37,50].

Autophagy in cancer is linked to the invasion process and also to therapeutic resistance. In PE, the autophagy impairment leads to shallow spiral artery remodeling, defective trophoblast invasion and increased apoptosis, followed by placental ischemia [37,49,50].

Tumor suppressor p53-binding protein 2 is upregulated in PE, is linked to enhanced autophagy and is associated with placental ischemia [37,50].

In summary, similar signaling pathways are used in both diseases, but cancer successfully uses autophagy for survival and adaptation, while preeclampsia is associated with breakdown of the autophagy–lysosomal system followed by placental dysfunction [37,48,49,50].

Immune cells are involved in both the placenta and in cancers, but their role has yet to be fully elucidated. Natural killer cells, T lymphocytes, macrophages, and regulatory T cells participate in antigen recognition, regeneration, and inflammation, crucial for maternal–fetal tolerance. They are also implicated in impaired antitumor immunity [51,52]. Autophagy inhibition leads to decreased uterine NK cell adhesion, contributing to miscarriage and PE [53]. On the other hand, autophagy is described as an important regulator of the immune TME [1,54]. Autophagy actively participates in the immune evasion of NK cells in cancer [1].

LC3 and Beclin-1, protein markers of autophagosomes, indicate autophagy activation in trophoblasts [55], but their role in vascular remodeling remains elusive. During the early phases of different cancers, autophagy exerts anti-malignant activity [56], limiting the production of anti-DNA damage through, e.g., ROS, and eliminating oncogenic proteins [57]. Autophagy also plays a key role in promoting cancer cell senescence in response to stress [1]. On the other hand, it can interact with pathways involved in cell metastasis and invasion [1].

In the placenta, autophagy plays a role in trophoblast development; however, in excess, it can lead to placental damage and ischemia. In cancer, it plays a dual role: in the early stage of tumor development, autophagy has anti-malignant functions and plays an important role in protecting the host tissue; in advanced cancer stages, it supports tumor metastasis and invasion [58].

However, major differences can be observed between placental development and tumor progression. Trophoblast cells follow an organized pattern of proliferation and invasion, while cancer is characterized by tumor cell spread and unorganized invasion [59,60]. Finally, the precise role of autophagy in vascular remodeling during cancer progression or placental development needs to be further studied. Some of these observations are presented below (Table 1).

## 5. Nanotechnology

Targeting autophagy is a promising therapeutic strategy [1]. The currently available drugs that target autophagy have limited clinical use because of their poor bioavailability and notable side effects. To overcome these issues, novel approaches such as custom-designed nanoplatforms (NPs) are warranted, including the use of machine learning technology [10,61,62].

Smart NPs are designed according to tumor location and characteristics in order to maximize success against cancer. Among these NPs, carriers with a size of 10 to 100 nm and a rod-like or spherical structure are the most efficient [7,63,64,65].

### 5.1. Mechanisms

Nanocarriers have the ability to encapsulate multiple drug molecules at a very small scale, enhancing the delivery of poorly soluble drugs and overcoming CSCs’ resistance mechanisms [30,38,63]. Particles smaller than 100 nm are favored in cancer therapy because this size optimizes the efficient accumulation in tumors [66]. Tumor blood vessels are disorganized, with gaps between endothelial cells smaller than 100 nm, which allow particles smaller than 100 nm to extravasate through these gaps from the blood into the tumor, avoiding filtering in healthy tissues. Also, these particles avoid immune clearance by the Reticuloendothelial System. Particles even smaller, such as under 20 nm, can penetrate deeper into the tumor parenchyma and are not immediately filtered by the kidneys [66].

Nanocarriers also possess improved tissue distribution and half-life circulation times, which decreases toxicity [7,30,67,68]. NPs may enhance the characteristics of therapeutic drugs, such as their stability, solubility, and accumulation in tumors [2]. Nanoparticles (NPs) are carriers that protect the active drug from degradation and control the drug release at the target site [69].

There are two types of mechanisms: passive and active targeting. The passive targeting enhances permeability and retention effects, based on how drugs are concentrated in the tumor rather than normal tissues [2,10,11,70]. This is due to the lack of adequate lymphatic outflow in the TME [11]. In active targeting, NPs can be conjugated with aptamers, antibodies, peptides, and other small molecules to enhance drug delivery [39,61]. Nanoparticles are internalized through various forms of endocytosis. The most common pathway is receptor-mediated endocytosis. After NPs bind to receptors, the particle is enveloped by a membrane, forming the endosome. Another way of entering cells is through micropinocytosis, which is an active process where cells take in a large amount of fluid and particles. After entering cells, the drug is released, because of endosomes, through osmotic swelling, membrane rupture, or by enzymes. Nanoparticles do not always need to enter the cell; they can act from the outside [70]. In this situation, after surface binding of NPs, the drug is released in the tumor interstitial space, which then diffuses into tumor cells. Another way is to change membrane curvature, which may lead to cell damage and death. Also, the tumor microenvironment (TME) can trigger drug release before entering the cells, directly killing tumor cells [71,72].

There are five steps for drug delivery system efficacy: blood circulation, tumor accumulation, tumor penetration, cellular internalization, and drug release [63,73]. In order to promote drug availability, nanoparticle surface modification is required, which also enhances targeted efficacy and reduces toxicity [10,74]. Innovative stimuli-responsive biomaterials have emerged for enhancing anticancer molecule bioavailability, with lower systemic toxicity and wider biodistribution [74]. To enhance their efficacy, stimulation-sensitive components have been added to different types of NPs such as metal–organic NPs, liposomes, and polymer micelles [2,7,75].

Smart NPs made of inorganic materials (such as gold) have been designed, while others use organic materials. The efficiency of NPs made with inorganic materials (such as gold) is better than that of those with organic materials [1,7,63]. Key advantages of these nanoparticles include high surface-to-volume ratios for drug loading, and the ability to improve bioavailability and cross epithelial fenestrations [76].

We provide the classification of the nanocarriers.

### 5.2. Nanoparticles Classification

#### 5.2.1. Based on Their Shape and Dimension

Different shapes and dimensions of NPs are developed in order to increase their ability to cross biological barriers:

Zero-Dimensional, where all dimensions are at the nanoscale (<100 nm), is used in cancer-targeted therapy, with different shapes: spheres, quantum dots, and fullerenes.

One-Dimensional, in which two dimensions are at the nanoscale, one is outside, for photothermal therapy: nanotubes, nano-worms, or nanorods.

Two-Dimensional with one dimension at the nanoscale, two outside used in bio imaging: nanofilms, graphene.

Three-Dimensional: Not confined to the nanoscale in any dimension, which are complex nanostructures [77].

#### 5.2.2. Classification by Composition

Organic Nanoparticles: liposomes, which are highly biocompatible, polymers, dendrimers, and exosomes [78]. Peptide-based vaccines have emerged as a powerful but safe neoadjuvant immunotherapy that targets specific proteins [79]. Furthermore, poly(lactic-co-glycolic acid nanoparticles were developed as a drug delivery system for chemotherapy, which enhanced their tumor suppression efficacy [80]. Lipid-based nanoparticles have been shown to enhance drug bioavailability and decrease side effects [81]. Polymeric micelles, with their high stability, increased bioavailability, and biodegradability, are widely used in oncology [82]. Hyaluronic acid can be conjugated with polyethylenimine to enhance stability [83]. Recently, extracellular vesicles (EVs) were investigated as a potential drug delivery system [84]. EVs are cell-derived nanostructures that are particularly important in cell-to-cell communication [85]. Some of these carriers, such as dendrimers, have catalytic effects of their own [79,82].

Inorganic Nanoparticles: metallic (gold, silver, iron oxide), quantum dots (crystals), and ceramic (silica) [77]. Gold nanoparticles can carry high drug doses but have the potential side effect of heating surrounding normal tissues [39,61,81]. Silica, silver or iron oxide NPs have catalytic effects of their own and have been used as autophagy inducers [79].

Carbon-based Nanoparticles: graphene and carbon nanotubes [77]. However, researchers observed CSCs’ high potential to avoid agent delivery [38]. Carbon-based NPs, specifically carbon nanotubes, are employed as carriers due to their nano-size, capacity to enter cells, and multifunctional capacity [25].

Hybrid Nanoparticles: Combinations of organic-inorganic or lipid-polymer [77].

#### 5.2.3. Classification Based on Synthesis Methods

Nanoparticle production is divided into two main approaches:

Top-down approach: based on breaking down bulk materials into smaller pieces through physical methods such as laser ablation.

Bottom-up approach, which means assembling atoms or molecules to form nanoparticles through chemical methods, for example, biosynthesis [78].

The heterogeneity that exists among human individuals is a major concern that must be taken into account [10,85,86]. Nanotechnology has been employed to target autophagy [2,11]. With their small size, NPs are taken up by cells, leading to autophagy through hypoxia and intracellular oxidative stress [2]. NP-induced autophagy is a possible cellular defense mechanism against NP toxicity [2].

Although autophagy-based nanotechnology seems feasible [79], controversies have arisen regarding whether to enhance or inhibit the process, depending on the specific role that autophagy plays in tumor development. However, strategies that stimulate autophagy are preferred [2,86]. Meanwhile, in other situations, autophagy has the exact opposite effect [87,88].

## 6. Autophagy-Targeting Treatment Options in Preeclampsia

Different strategies have been sought to improve angiogenesis in the placenta, one of them being autophagy targeting. In this regard, Cyclosporin A has been evaluated as a potential treatment option in PE; it showed good results in stimulating autophagy, and its immunosuppressant effect is benign and has no side effects on trophoblasts [89].

Another therapeutic avenue is using proton pump inhibitors such as Esomeprazole. Stimulated by L-NAME through hypoxia, autophagy was induced in mice with elevated blood pressure, inhibiting mTOR. Esomeprazole activates mTOR, inhibiting L-NAME-induced autophagy and potentially ameliorating PE [13]. Esomeprazole also suppressed hypoxia by reducing HIF-1α protein levels [13]. This treatment is safe and well-tolerated during pregnancy. It also decreases circulating concentrations of sFlt-1 and soluble endoglin [90,91,92,93].

Targeting protein degradation using the autophagy–lysosomal pathway is a potential therapeutic avenue. PE is characterized by the excessive accumulation of damaged proteins in the placenta [81]. Toxic protein aggregate accumulation can be inhibited by restoring autophagy. This targeted treatment was used in Alzheimer’s disease, but similar protein aggregation was observed in PE [63,94,95]. Lysosomal activity can be targeted by trehalose, which is an autophagy activator. Trehalose, a disaccharide, induces autophagy and inhibits protein-aggregation, acting as a chaperone and protecting against PE-associated hypoxia [63,96,97].

### Nanotechnology in Autophagy-Targeted Strategies in PE

The introduction of nanotechnologies in targeted therapy has improved the safety and efficacy of therapeutics [67]. Black phosphorus is a two-dimensional nanomaterial that causes apoptosis in a dose-dependent manner. It has high biodegradability and biocompatibility but exerts high toxicity [98].

Targeting oxidative stress is one of the most accepted strategies in PE. Pravastatin already demonstrated its efficacy in enhancing VEGF-like angiogenic factor (PlGF) expression [15,99] and reducing preeclampsia manifestation [90]. Metformin reduces ROS through activated protein kinase (AMPK) activation [100,101]. Combined with esomeprazole, it reduces sFlt-1 secretion [102], demonstrating good results in PE. Resveratrol has shown similar effects [103]. Melatonin reduces ROS, modulating autophagy without reducing sFlt1 [15,104].

Levo-arginine is used to enhance angiogenesis and can be uploaded via NPs [105]. Mesenchymal stem/stromal cell (MSC)-derived EVs are nanoplatforms for carrying antioxidant enzymes and miRNAs that reduce ROS levels in the placenta [106]. This treatment gained interest for treating PE, as it was already being used in several pathologies such as Alzheimer’s disease, rheumatoid arthritis or stroke [15]. It exerts antioxidant activity that not only reduces sFlt-1 but also TNF-α and IL-6 expression, exhibiting anti-inflammatory effects [15].

Mitochondria-targeting therapy using Coenzyme Q10 could be a promising treatment for preeclampsia [107]. MitoQ is another drug with antioxidant effects that inhibits mitochondrial activation [15,108]. AP39 is a mitochondria-targeting hydrogen sulfide donor which, as Hu et al. demonstrated, reduces HIF-1α protein expression [9,109].

Zhu et al. developed folic acid-modified lipid nanoparticles (FA-LNPs). This nanoplatform encapsulated FALNP@si-PKM, a small interfering RNA targeting pyruvate-kinase M with significant importance in PE management [110]. Placental injury can be targeted using 6-gingerol, which has a protective effect in PE [64].

NPs were developed to target oxidative stress and autophagy. This technology is challenging, as NPs have poor solubility, limited permeability and rapid metabolism, which reduces their effects [111]. To maximize efficacy while minimizing adverse effects, optimal drug combinations have been explored [15]. However, nanotechnology offers significant advantages, including the possibility to target specific cells/tissues of interest [65].

## 7. Targeting Autophagy in Cancers

Several drugs have been employed to target autophagy in cancers, such as chloroquine (CQ) and hydroxychloroquine (HCQ). Although these drugs were developed for other purposes, they can activate or inhibit autophagy [2]. Autophagy may preserve the characteristics of CSCs, so inhibiting this process with Chloroquine (CQ) and Bafilomycin may lead to cells’ self-renewal ability being decreased [1].

Chemotherapy is a treatment specific to cancer with significant side effects [112]. These challenges may be overcome through NP systems, with their enhanced solubility and stability [2]. Chemotherapy can itself induce mild autophagy, thus protecting tumor cells and inducing chemoresistance. Furthermore, drug accumulation in the tumor microenvironment (TME) and at the tumor site is essential, and this can be achieved with new nanoparticles developed to target autophagy [113,114]. The tumor microenvironment (TME) can be changed using stimulus-responsive nanocarriers, such as amphiphilic polymers, which can improve drug therapeutic effectiveness [115,116]. Researchers have developed an amphiphilic polymer conjugated with doxorubicin, in combination with the autophagy inhibitor LY294002. This NP changed macrophage immunological clearance [2,115].

Chen et al. used metal–organic frameworks as delivery nanoplatforms in tumor treatment for the autophagy inhibitor 3-methyladenine (3-MA). This NP has a high porosity and a large surface area for enhanced drug delivery [117].

Phototherapy has also been used to modulate autophagy. It is based on thermal-sensitive materials, which can convert light energy into heat to destroy tumors [81,118]. This process may lead to the production of autophagy-inducing ROS [2,119]. Zhang et al. developed organosilica nanodots, which are associated with photosensitizer tetraphenylporphinesulfonate; they constitute an autophagy-targeted therapy that acts against the lysosome [120]. In the same way, iron oxide NPs, in combination with the autophagy inhibitor chloroquine CQ created by Ren et al., can be used for cancer treatment [121]. Hyperthermia can be combined with autophagy metallic nanoparticles to kill tumor cells based on ROS production [10,79]. Metallic nanoparticles not only allow phototherapy to obtain heat, but also ionizing radiation, laser, or microwaves [10,122]. Magnetothermal effects were achieved by Wang, who developed a novel NP—a mesoporous magnetic copper ferrite nano-agent—which accelerates ROS production. This NP was uploaded with the autophagy inhibitor chloroquine, which inhibits cancer cells’ resistance to hyperthermia and oxidative stress [123]. Autophagy modulation can be obtained using a chaperone required for collagen maturation, such as heat shock protein 47, and has therapeutic effects [10,124].

The primary obstacle in cancer treatment is the TME, which is characterized by hypoxic conditions and increased autophagy. This process can promote cancer cell survival, especially CSCs, in a dormant state. To address this issue, researchers have developed nano-diamonds (NDs), which are atypical autophagy inhibitors [79]. Anti-angiogenic agents can be uploaded to enhance this process [125]. Furthermore, the downregulation of HIF-1*α* expression in tumor cells can effectively inhibit tumor metastasis [79,126]. In the tumor microenvironment (TME), autophagy plays a crucial role in dormant CSC regeneration. Tumor recurrence and metastasis could be overcome through autophagy inhibition in the TME [127]. Different strategies are developed to modulate the TME: NPs such as vaccines, immune checkpoint blockade, and autophagy inhibitors combined with chimeric antigen receptor T cells, which act as immunomodulators [2,128]. ROS accumulation also leads to Fenton reactions between Fe^2+^ and H_2_O_2_, and ferroptosis is followed by tumor cell death [2,129].

The most powerful autophagy repressor is pharmacological mTOR inhibition [10,79,130]. Nanoplatforms have been used to deliver the autophagy inducer, rapamycin. Yan et al. made an NP based on polymeric micelles for this purpose, in combination with the antitumor drug 9-Nitro-20(S)-camptothecin [11], while Yan et al. used hyaluronic acid as a carrier for the same drug combination. In this sequencing, the autophagy inducer rapamycin is released earlier for efficient autophagy, followed by 9-NC. As such, tumor cells’ sensitivity to chemotherapy is enhanced [73]. Different pathways are required for drug uptake, such as that of lysosomes, which create the conditions for autophagy [74]. In this regard, hyaluronic acid (HA) is used to target tumor cells through CD44 receptor-mediated endocytosis [73]. Impaired autophagy is followed by the accumulation of damaged mitochondria, which release ROS; it also leads to genetic mutations and cellular organelle degradation, leading to type II cell death. DNA damage is followed by malignant transformation and chronic inflammation [131,132]. The autophagy inhibitor 3-methyl-adenine (3-MA) enhances the antitumor effects of immunotherapy, such as that with trastuzumab (Tmab), an HER2-specific monoclonal antibody [10,133].

Chloroquine (CQ) and hydroxychloroquine (HCQ) are the FDA-approved compounds that modulate autophagy [122]. HCQ enhanced the efficacy of mTOR inhibitors like everolimus [134,135], which has been approved by the FDA as an antiangiogenic compound [136]. However, its high resistance rates reduce its clinical utility [137]. To enhance their efficacy, chemotherapy was combined with HCQ in a clinical trial (NCT01206530) [10].

Autophagosomes, which include depolarized mitochondria, represent an important step in autophagy [138]. Nanomaterials developed to target this specific process improve the sensitivity of tumor cells to chemotherapeutic agents [139]. It has been observed that autophagy can suppress T cell-mediated antitumor immunity, which is a key component of tumor immune escape [140]. This process can be overcome using immunotherapy [28]. A new nanoparticle has been designed to remodel the TME and double hydroxide nanoparticles while enhancing tumor immunotherapy [141].

Other autophagy-targeted nanocarriers have been tested in combination with chemotherapy, for example, hybrid dendritic–linear–dendritic block copolymers (HDLDBCs) as NPs loaded with Chloroquine and chemotherapy against autophagosomes. Fe3O4 magnetic NPs were combined with chitosan chloride to target lysosomes, while albumin nanoparticles were combined with Rapamycin to target mTOR [142]. Albumin-bound rapamycin (ABI-009) nanoparticles were tested in several clinical trials on cancers [143]; they did not show any toxicity and also strongly inhibited mTOR targets [10].

We already emphasized the importance of gold nanoparticles because of their size and surface [144]. Other metal-based NPs, such as iron and copper oxide nanoparticles, have also been tested; they induce oxidative stress, which leads to autophagy via AMPK/mTOR pathway dysregulation followed by mitochondrial damage [145].

Nucleic acids can be used to regulate autophagy-related genes (ATGs); in such situations, we can use microRNAs (miRNAs), small interfering RNA (siRNA), plasmid DNA (pDNA) or aptamers [146]. Their high affinity for the target and low immunogenicity make them suitable for targeted treatment [147]. Beclin-1, which is used to target ATG6, is the most widely studied [79]. ATG4 can be targeted by miRNA-34 [144], the effectiveness of which has also been tested against ATG5 [148] and ATG9 [149]. As NPs, superparamagnetic iron oxide nanoparticles (SPIONs) were preferred, for example, those linked to the autophagy inhibitor miRNA-376B [150]. In Table 2, we summarize the autophagy modulation-based treatment options in preeclampsia and cancers.

## 8. Mutual Reference and Translational Applicability of Nanomedicine Research Strategies

By identifying common mechanisms and observing differences in molecular patterns in preeclampsia and cancers, future directions in therapeutic strategies are emerging. Cellular behavior within microenvironments is strongly influenced by spatial and temporal gradients, as well as local transport phenomena. In cancer therapy, the primary goal is to inhibit survival in cancer cells, while in placental impairment, it is to restore homeostasis. We observed that, in cancers, autophagy is usually overactivated but has a dual role, while it is impaired in preeclampsia. Nanotechnology is used in cancer as an autophagy inhibitor or activator; in preeclampsia, we use NPs to restore autophagy. As we have highlighted, and as is obvious in the tables presented here, in cancers, the key mechanism of autophagy modulation through NPs is ROS elevation and lysosome inhibition. In PE, nanomedicine is used to improve nutrient and energy supply. NPs largely target the TME in cancers. In PE, nanotechnology is used to target trophoblast trafficking.

While both conditions, cancer and PE, involve aggressive cellular behavior associated with impaired angiogenesis, cancer therapy strategies can be translated into preeclampsia interventions. The field of nanomedicine holds significant promise for targeted drug delivery to prevent off-target toxicity. Because preeclampsia could be considered very similar to a tumor behavior due to the invasive nature of trophoblasts accompanied by abnormal placental vascular remodeling, nanotechnology used in cancer treatment, such as lipid nanoparticles, can be adapted to treat the placental damage [151].

### 8.1. Shared Nanomedicine Research Strategies and Design Paradigms

Homing Peptides: Peptides initially used to target tumor neo-vasculature, such as CGKRK, have shown potential to also target the uterine placental vasculature.

Nanoplatforms Drug Delivery: Different carriers, such as lipid nanoparticles, are designed to target the placenta and enable the down-regulation of anti-angiogenic factors without affecting the fetus [83,151].

Translational approaches from cancer treatment show that active targeting mechanisms, ligand-mediated, can be combined with passive, enhanced permeability and retention (EPR)-like behavior to improve drug accumulation in the placenta [83,151].

Transplacental passage of drugs to the fetus could be overcome by nanocarriers, protecting the fetus while treating the placenta. In a similar way, nanocarriers protect normal tissue from chemotherapy, which is kept within the tumor site [7,83,151].

### 8.2. Translational Applicability and Mutual Reference

While pregnancy and cancer share similar behavior, potential interchange of target delivery should be searched. ROS-responsive nanoparticles used in cancer therapy have been adapted to target the placenta to inhibit the release of toxic factors in preeclampsia. Meanwhile, aiming to enhance the therapeutic effect while also limiting the fetal injury [48,152].

We face key differences and challenges. The most important difference consists of high mortality associated with cancers, in which cellular invasion, proliferation and metastasis are uncontrolled and chaotic, while the placenta is a highly specialized organ in which proliferation and invasion are limited in time and space [48,49,152].

Further research is focused on developing multifunctional nanoplatforms for both early diagnosis and targeted therapy of placental dysfunction, based on cancer treatment strategies [152,153].

## 9. Conclusions

Some of the cellular processes required for proper placentation, including autophagy, are common to both placental development and tumor progression. Just as trophoblast cells invade and migrate, so do cancer cells. While in the trophoblast, this phenomenon is programmed and controlled; in cancer, any such regulation is lost. We thus observed that autophagy has a dual role in cancer; as we have shown, it can act as a tumor promoter or inhibitor, depending on the stage. A better understanding of the similarities and differences between the role of autophagy in placentation and cancers could lead to the development of effective targeted therapies.

Autophagy-based nanotechnology seems to be a feasible option in both placental ischemia in preeclampsia and cancers. This review draws parallels between targeted treatments in malignancies and placenta-derived PE. Comparing these diseases provides a novel molecular rationale and helps us identify novel autophagy-modulating treatments.

## Figures and Tables

**Table 1:** Characteristics of autophagy in preeclampsia and cancers.

Autophagy Stimulation Mechanism	Triggers in PE	Triggers in Cancers
Environmental hypoxia	ROS [15,48,49]	
	Pro-inflammatory factors and immune cells [18,19];	
	Damaged mitochondria [20,21,22,23] and mitochondrial DNA damage [24,25,26]; HIF-1α and LC3-II activation [13,31,32,33,34,35,48] VEGF protein expression [37].	HIF-1α [47,48] Suppression of the mTOR pathway and activation of AMPK [47,48]
Autophagy-related gene	(mTOR) complex [12,16,17];	
	PI3K/AKT/mTOR [27,28,29]; p53 [1]; BNIP3 expression [38]; PKM, LEP, and HK2 expression [39].	
TME	Immune cells [51,52]	HIF [42,43];
		VEGF and FGF [1,46]; Immune cells [1,51,52,53,54]; LC3 and Beclin-1, protein markers of autophagosomes [56,57]. Immune cells [1,53,54]

ROS—reactive oxygen species; HIF—hypoxia-inducible factor; LC3-II—light chain protein; VEGF—vascular endothelial growth factor; FGF—fibroblast growth factor; TME—tumor microenvironment.

**Table 2:** Autophagy modulation-based treatment options in preeclampsia and cancers.

Autophagy Modulation	Therapy	Mechanism
Preeclampsia	Proton pump inhibitors [13]. Cyclosporin A [89].	Immune-suppressant effect. Inhibiting mTOR
	Proton pump inhibitors [90,91,92]. Targeted protein degradation, trehalose [63,93,94,95,96,149]. Pravastatin [15,98]. Metformin [99,100], resveratrol [102], melatonin [103], EVs [105], Coenzyme Q10 [106], MitoQ [108], AP39 [108] FALNP@si-PKM [109].	Reducing HIF-1α, sFlt-1 and soluble endogline. Autophagy–lysosomal pathway. Targeting oxidative stress. Mitochondria-targeting therapy. RNA-targeting pyruvate-kinase M.
Cancers	Chloroquine (CQ), Hydroxychloroquine (HCQ) [1,2,150].	Activate or inhibit autophagy, enhance the mTOR-inhibiting effect.
	Autophagy inhibitor LY294002 on NPs [2,110]. Phototherapy [2,118,119,120]. Autophagy metallic nanoparticles with phototherapy [10,79,121,123]. Nano-diamonds [79]. Anti-angiogenic agents [124,125,126,127]. Rapamycin [10,79,129]. Hyaluronic acid as NPs [73]. Autophagy inhibitor 3-methyl-adenine [132]. Nanomaterials developed to target autophagosomes [136,137,138,139,140,141]. Metal-based NPs [143]. Beclin-1 [79]. miRNA-34 [145,146,147,148]. Iron oxide nanoparticles (SPIONs) [148].	Immunological clearance by macrophage. ROS production, which induces autophagy. Autophagy through heat shock protein 47. Autophagy inhibitors. HIF-1*α* expression downregulation. mTOR. Enhance trastuzumab effect, enhance immunotherapy. Mitochondrial damage. ATG6, ATG4, ATG5 and ATG9. Autophagy inhibitor miRNA-376B.

## Data Availability

Data are available from the corresponding author upon request.

## Abbreviations

The following abbreviations are used in this manuscript:

EMT	Epithelial-to-mesenchymal transition
TME	Tumor microenvironment
CSCs	Cancer stem cells
ROS	Reactive oxygen species
mtDNA	Mitochondrial DNA
PE	Preeclampsia
Atg	Autophagy-related gene
mTOR	mammalian target of rapamycin
PI3K	Phosphatidylinositol-4,5-bisphosphate 3-kinase
HIF	Hypoxia-inducible factor
LC3	Light chain protein
VEGF	Vascular endothelial growth factor
FGF	Fibroblast growth factor
NPs	Nanoplatforms
EVs	Extracellular vesicles
AMPK	Activated protein kinase
PlGF	Placental growth factor
sFlt1	FMS-like tyrosine kinase-1
FA-LNPs	Folic acid-modified lipid nanoparticles
CQ	Chloroquine
HCQ	Hydroxychloroquine
